# Cost-effectiveness of open versus laparoscopic pancreaticoduodenectomy: a retrospective Markov model analysis from China

**DOI:** 10.3389/fonc.2025.1616793

**Published:** 2025-12-17

**Authors:** Yan Zhu, Qi Liu, Hua Cheng, Xiaoding Shen, Ziyao Wang, Danmei Liang, Nengwen Ke, Xiaoxia Zhang

**Affiliations:** 1 West China School of Nursing, Sichuan University, Chengdu, China; 2 Mental Health Center, West China Hospital, Sichuan University, Chengdu, China; 3 Intensive Care Unit, West China Hospital, Sichuan University, Chengdu, China; 4 Division of General Surgery, Shang Jin Hospital of West China Hospital, Sichuan University, Chengdu, China; 5 Division of Pancreatic Surgery, Department of General Surgery, West China Hospital, Sichuan University, Chengdu, China; 6 Cancer Center, West China Hospital, Sichuan University, Chengdu, China; 7 Innovation Center of Nursing Research, Nursing Key Laboratory of Sichuan Province, West China Hospital, Sichuan University, Chengdu, China

**Keywords:** cost-effectiveness, Markov model, laparoscopic pancreaticoduodenectomy, open pancreaticoduodenectomy, pancreatic ductal adenocarcinoma

## Abstract

**Introduction:**

Open pancreaticoduodenectomy (OPD) and laparoscopic pancreaticoduodenectomy (LPD) are the two main surgical approaches for treating pancreatic cancer.

**Objective:**

To evaluate the cost-effectiveness of OPD and LPD in treating pancreatic ductal adenocarcinoma by establishing a Markov Model.

**Methods:**

Patients with pancreatic ductal adenocarcinoma who staged at I-III undergone OPD or LPD were retrospectively included from March 2017 to December 2020. Patients were followed up by telephone until June 2024. A Markov Model was established to simulate disease progression after 120 cycles by including survival data and average hospitalization costs.

**Results:**

Two hundred patients were included, 100 for OPD group and the other 100 for LPD group. The results indicated that after 10 years of model operation, the LPD group had an increased cost of 13,175.31 yuan compared to the OPD group, with an incremental effect of 0.063 per quality-adjusted life years (QALY). It also showed that the incremental cost-effectiveness ratio value was ¥205,864.22per QALY, which was less than willing to pay (¥268,074.00).

**Conclusion:**

This study is the first to analyze the cost-effectiveness of OPD versus LPD in PDAC. The study indicated that LPD remains an acceptable operation with certain cost-effectiveness for pancreatic cancer patients. However, due to the low survival rates and the fact that LPD is a highly technique-dependent operation for pancreatic cancer, surgeons should keep cautious of the choice between the OPD and LPD based on the patient’s expectation and financial situation.

## 1 Research introduction

According to the 2024 data from the International Agency for Research on Cancer (IARC), pancreatic cancer incidence and mortality rates continue to rise, with 510,566 new cases and 467,005 deaths reported (1). The latest data released by the National Cancer Center (NCC) of the Chinese Academy of Medical Sciences reported approximately 134,374 new cases and 131,203 deaths in China in 2022, ranking pancreatic cancer eighth in overall incidence and sixth in mortality among all cancer types (2). Research suggests that by 2030, pancreatic ductal adenocarcinoma (PDAC) will become the second leading cause of cancer-related deaths worldwide, surpassing liver and other solid tumors (3), posing a severe threat to human health. Pancreatic cancer is highly malignant, with occult onset, non-specific early clinical symptoms, and strong invasiveness (4). Approximately 50% of patients have metastatic disease at the time of initial diagnosis, with an overall 5-year survival rate of less than 13% (5, 6).

The high treatment costs are particularly concerning given the low survival rates associated with pancreatic cancer. Research estimates that by 2050, the global economic expenditure on pancreatic cancer treatment will reach 129 trillion US dollars, accounting for 0.28‰ of the per capita Gross Domestic Product (GDP) (7). Studies have shown that in 2017, the hospitalization costs for cancer treatment in China had already reached 304.80 billion yuan, of which the hospitalization costs for pancreatic cancer accounted for 5.29 billion yuan (8). The enormous medical expenses impose a severe burden on the socio-economy, exacerbate the financial situation of patients’ families, and adversely affect patients’ treatment compliance and disease prognosis.

Surgical resection is the preferred method for treating pancreatic cancer. The traditional surgical approach is open pancreatoduodenectomy (OPD). In 1994, Gagner and Pomp reported the world’s first laparoscopic pancreatoduodenectomy (LPD) (9, 10). Since then, minimally invasive surgical methods represented by LPD have been increasingly applied in the field of pancreatic surgery, accounting for about 20% of all pancreatoduodenectomies by 2021 (11). In 2017, the pancreatic surgery group, represented by the Pancreatic Surgery Group of the Surgical Branch of the Chinese Medical Association, issued an expert consensus on laparoscopic pancreatoduodenectomy, standardizing the implementation of the LPD procedure and further promoting the development of LPD surgery (12). There have been numerous studies comparing the advantages and disadvantages of the two surgical methods, but most of these studies compare the early complications and side effects, as well as the medium and long-term survival rates (13–16). Currently, there is a lack of literature analyzing and comparing the economic effects of the two surgical methods.

Health economic evaluation is a branch of economic evaluation that can comprehensively evaluate the cost of obtaining unit effects by integrating both inputs (costs) and outputs (effects). Health economic evaluations support the health care decision-making process by providing information on costs and consequences of health interventions. They can provide useful information to policy makers, payers, health professionals, patients, and the public about choices that affect health and the use of resources (17, 18). Given the low survival rate and high treatment costs of pancreatic cancer, particularly in resource-limited settings, an economic evaluation of OPD versus LPD is warranted. Therefore, this study will establish a Markov model based on patient information from a national regional medical center in China, using cost-effectiveness analysis to evaluate the health economic benefits of OPD and LPD in treating pancreatic cancer, providing clinical evidence on cost-effectiveness for doctors and patients when choosing surgical methods.

## 2 Materials and methods

### 2.1 Data source

This study retrospectively collected patients who were biopsy-confirmed with pancreatic cancer and underwent radical surgery for pancreaticoduodenectomy at the pancreatic surgery department of a national regional medical center in Western China from March 2017 to December 2020. Patients were divided into OPD and LPD groups based on the surgical approach, and followed up regularly by telephone until June 2024. This study has been approved by the Biomedical Ethics Committee of the research institution (number: 2024-9–13 NO: 1689).

### 2.2 Inclusion and exclusion criteria for study subjects

#### 2.2.1 Inclusion criteria

(i) Patients diagnosed with pancreatic ductal adenocarcinoma through histological or cytological examination, with pancreatic cancer staging according to the 8th edition of AJCC TNM staging I-III (19); (ii) Patients who have undergone radical pancreatoduodenectomy, including those with combined vascular resection; (iii) Age > 18 years; (iv) Predicted survival > 3 months; (v) No distant metastasis to the liver, lungs, or other organs.

#### 2.2.2 Exclusion criteria

(i) Patients who underwent total pancreatectomy; (ii) Patients with tumor diseases other than pancreatic ductal adenocarcinoma; (iii) Patients assessed as R1 resection postoperatively; (iv) Patients with other diseases that seriously affect survival, such as chronic kidney disease requiring dialysis, chronic obstructive pulmonary disease requiring home oxygen therapy, coronary heart disease of III-IV grade, etc; (v) Patients who died of non-tumor causes within 90 days postoperatively; (vi) Subjects with low compliance who are unwilling to cooperate with the research follow-up.

### 2.3 Establishment of Markov model

This study categorizes the disease progression of pancreatic cancer into three health states: Disease-free survival (DFS), Progressed disease (PD), and Death (D). A Markov model was constructed to evaluate the economic aspects of two treatment strategies. All simulated patients begin in the DFS state, either maintaining their original DFS state or transitioning to the progressed disease state in the following month based on the transition probabilities. Patients in the progressed disease state can only transition between the progressed disease and death states. The Markov simulation cycle is set for 120 cycles (10 years), with each cycle lasting one month. At the end of each cycle, all patients enter the death state. Given the rapid progression of pancreatic ductal adenocarcinoma, employing a monthly cycle allows for more precise calculation of monthly state transition probabilities based on clinical survival data. A half-cycle correction was applied to account for the fact that transitions can occur at any point within a cycle, thereby improving the accuracy of the estimated costs and outcomes. The simulation duration was set at 10 years to encompass the disease endpoint status of virtually all patients. By the conclusion of the simulation, the majority of patients had reached the death state, thereby ensuring the stability and reliability of the results.

**Figure 1:**
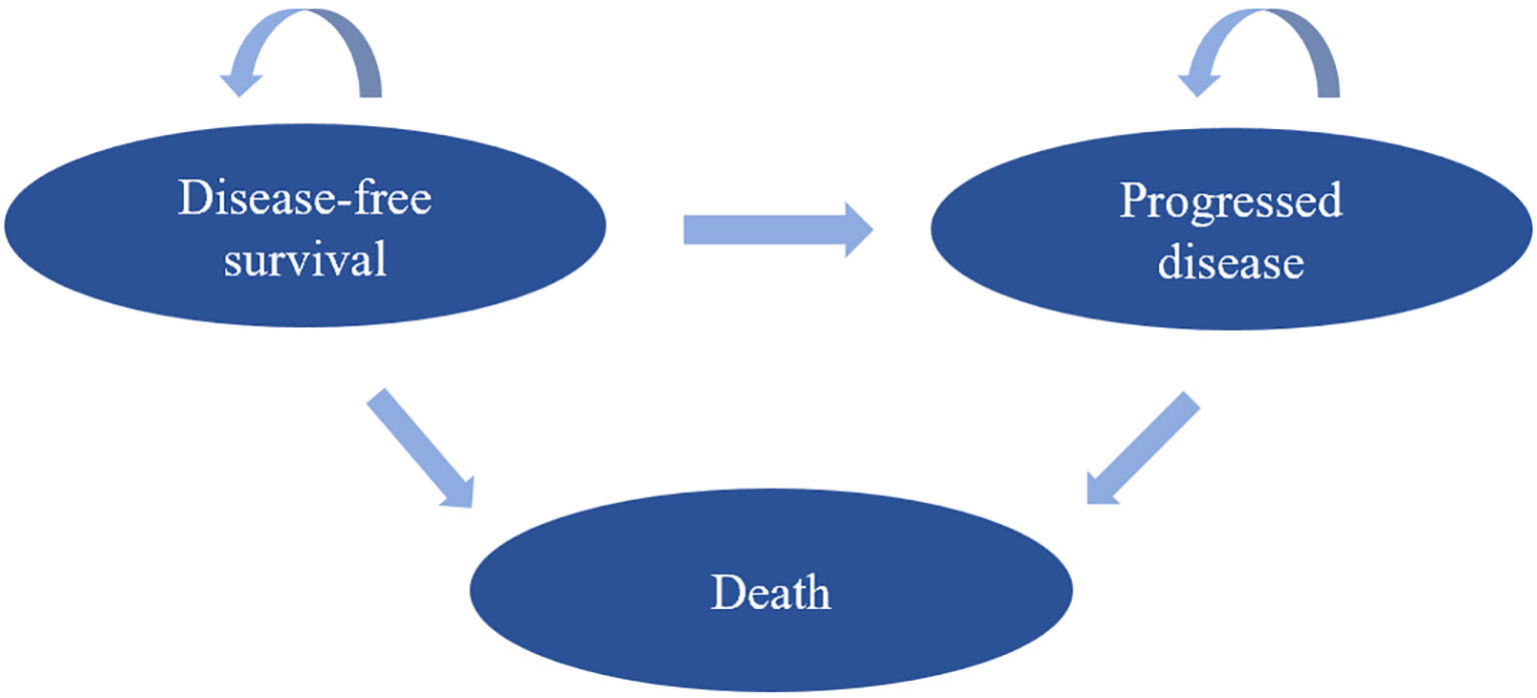
Markov state transition diagram. Arrows indicate a state that remains in its original state or transitions to another state.

We used the progression free and progression disease duration to estimate transition probabilities in the Markov model as following: Risk of an event (1 month) = [1-(0.5) ^ (1/median time to event)]. This can be easily derived through the equations: P = 1-e-R and R = -ln [0.5]/(time to event/number of treatment cycles) (20, 21). The Markov transition probabilities are presented in **Table 1**. The Markov state transitions are shown in **Figure 1**. The specific transition probability formula for the Markov model is shown in **Supplementary Figure S1**.

**Table 1:** Probability of state transition.

Transfer probability	Baseline value	Lower limit	Upper limit
Open pancreatoduodenectomy			
P dfs-dfs	0.921	0.737	1
P dfs-pd	0.048	0.038	0.058
P dfs-d	0.031	0.025	0.037
P pd-pd	0.917	0.734	1
P pd-d	0.083	0.066	0.100
Laparoscopic pancreatoduodenectomy			
P dfs-dfs	0.927	0.742	1
P dfs-pd	0.045	0.036	0.054
P dfs-d	0.028	0.022	0.034
P pd-pd	0.926	0.741	1
P pd-d	0.074	0.059	0.089

The transition probability of P dfs-dfs from DFS state to the next cycle DFS state. Lower limit: baseline value - baseline value × 20%. Upper limit: baseline value + baseline value × 20%. The upper limit does not exceed value of 1.

Dfs, disease-free survival; Pd, progressed disease; d, death.

### 2.4 Parameters of Markov model

The medical costs for patients with pancreatic cancer were obtained by querying the HIS system of the research institution. Medical costs include direct medical costs, indirect medical costs, and hidden costs. Direct medical costs mainly include hospitalization fees, surgery fees, examination and testing fees, treatment costs for postoperative complications, and follow-up costs; indirect medical costs include the loss of labor time and productivity for patients and their families due to illness, disability, or death, including losses caused by school suspensions, work leave, premature death, etc. Hidden costs mainly include the pain, anxiety, tension, and other physiological and mental suffering and discomfort caused by the disease or the implementation of preventive, diagnostic, and other medical services. Since indirect and hidden costs are inconvenient to collect and some costs are difficult to measure in monetary terms (22), this study only analyzes direct medical costs. The cost data for this research were obtained from the hospital information system. The data cover direct medical expenses from 2017 to 2020, including hospitalization fees, surgery and anesthesia costs, treatment costs for postoperative complications, outpatient follow-up fees, and medication costs. All costs were adjusted to 2023 constant prices using the healthcare Consumer Price Index (CPI) published by the National Bureau of Statistics of China (23). Previous research indicates that utility scores among Chinese and European populations, both in the general population and among cancer patients, are quite similar (ranging from 0.828 ± 0.184 *vs* 0.838 ± 0.154) (24, 25), suggesting no significant cross-country differences. Due to the current lack of utility data for Chinese patients undergoing pancreatic cancer surgery, this study adopted utility values from two well-established sources (26–28). According to the 2011 Chinese guidelines for pharmacoeconomic evaluation, the discount rate for this study is 5.00% (22). Model parameters are shown in **Table 2**.

**Table 2:** Model parameters of Markov.

Parametric	Open pancreatoduodenectomy	Laparoscopic pancreatoduodenectomy
Direct costs (RMB,2023 values)	95,994.18	100,013.20
Utility value		
DFS	0.792	0.810
PD	0.650	0.650
Discount rate (%)	5	5
Survival data		
Median DFS/months: (HR = 1.452; 95% CI = 1.051 - 2.006; P = 0.024)	14	15
Median OS/months: (HR = 1.310; 95% CI = 0.95-1.807; P = 0.100)	22	24

CI, confidence intervals; DFS, disease-free survival; HR, Confidence intervals; OS, over survival; PD, progressed disease.

The primary outcome indicators of this study are the incremental cost-effectiveness ratio (ICER). The utility values corresponding to each stage of the disease are represented by U; costs are represented in the form of expenses C (unit: yuan), where ICER = (C1 - C2)/(U1 - U2). According to the World Health Organization’s evaluation criteria, when the ICER is less than the willingness to pay (WTP), the treatment plan is considered cost-effective; when the ICER is greater than the WTP, the treatment plan is not considered cost-effective (22). This study adopts three times the 2023 per capita GDP of China as the WTP threshold, which is 268,074.00 yuan, to evaluate the optimal strategy under the set WTP.

### 2.5 Sensitivity analysis

#### 2.5.1 One-way sensitivity analysis

This study uses one-way sensitivity analysis to evaluate the uncertainty of model parameters, assessing the impact of one parameter on the results while keeping other parameters constant. The results are presented in the form of a tornado diagram.

#### 2.5.2 Probabilistic sensitivity analysis

This analysis method is used to evaluate the comprehensive impact of all model parameters on the cost-effectiveness results of the two strategies. The model is run 1,000 times in the form of Monte Carlo simulation, and the results are represented through a cost-effectiveness acceptability curve.

## 3 Results

### 3.1 Baseline characteristics of patients

A total of 200 patients were included in this study, with 100 patients in both the OPD and LPD groups. In both groups, there were more males than females. The majority of patients with locally advanced pancreatic cancer in both groups had pathological stages IB and IIB, with the tumor differentiation mainly consisting of moderately to poorly differentiated and moderately differentiated types. The specific baseline characteristics are shown in **Table 3**.

**Table 3:** Baseline information of OPD and LPD (N = 200).

Variable	OPD (n=100)	LPD (n=100)	t/*χ* ²	*P*
Age (M ± SD), n (%)	60.97 ± 10.70	63.82 ± 11.33	-17.263	0.220
Sex, n (%)			-3.809	0.382
Male	59	65		
Female	41	35		
Pathological stage, n (%)			-0.647	0.518
IA	10	11		
IB	49	46		
IIA	2	17		
IIB	39	26		
Grade of differentiation, n (%)			-0.506	0.613
Poorly differentiated	9	12		
Low-moderately differentiated	5	4		
Moderately-Low differentiated	45	46		
Moderately differentiated	36	33		
Moderately-highly differentiated	5	5		

LPD, laparoscopic pancreatoduodenectomy; M, mean; OPD, open pancreatoduodenectomy; SD, standard deviation.

### 3.2 Cost-effectiveness analysis

**Figure 2:**
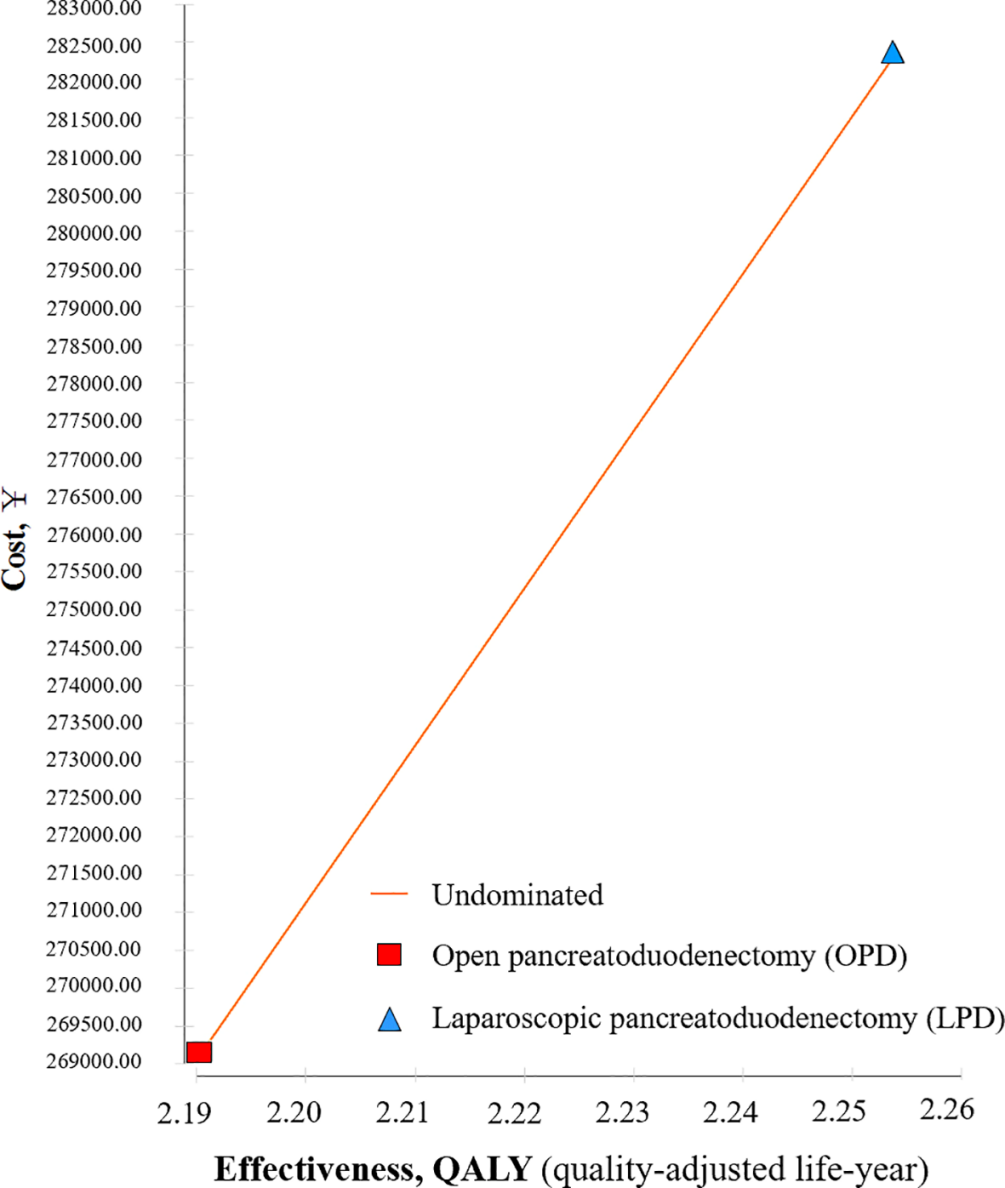
Cost-effectiveness analysis of OPD and LPD.

The results indicated that after 10 years of model operation, the LPD group had an increased cost of 13,175.31 yuan compared to the OPD group, with an incremental effect of 0.063 per quality-adjusted life years (QALY). The ICER value was 205,864.22 yuan/QALY, which is less than the WTP (268,074.00 yuan). Compared to the OPD group, the LPD group demonstrated a more cost-effective advantage, as shown in **Table 4** and **Figure 2**.

**Table 4:** Cost-effectiveness analysis of OPD and LPD.

Treatment strategies	Cumulative cost (RMB)	Effects (QALYs)	Incremental cost (RMB)	Incremental effects (QALYs)	ICER (RMB/QALYs)
OPD	269,164.88	2.190	—	—	—
LPD	282,340.19	2.254	13,175.31	0.063	205,864.22

ICER, incremental cost-effective ratio; LPD, laparoscopic pancreatoduodenectomy; OPD, open pancreatoduodenectomy; QALY, Quality-adjusted life years.

### 3.3 One-way sensitivity analysis

**Figure 3:**
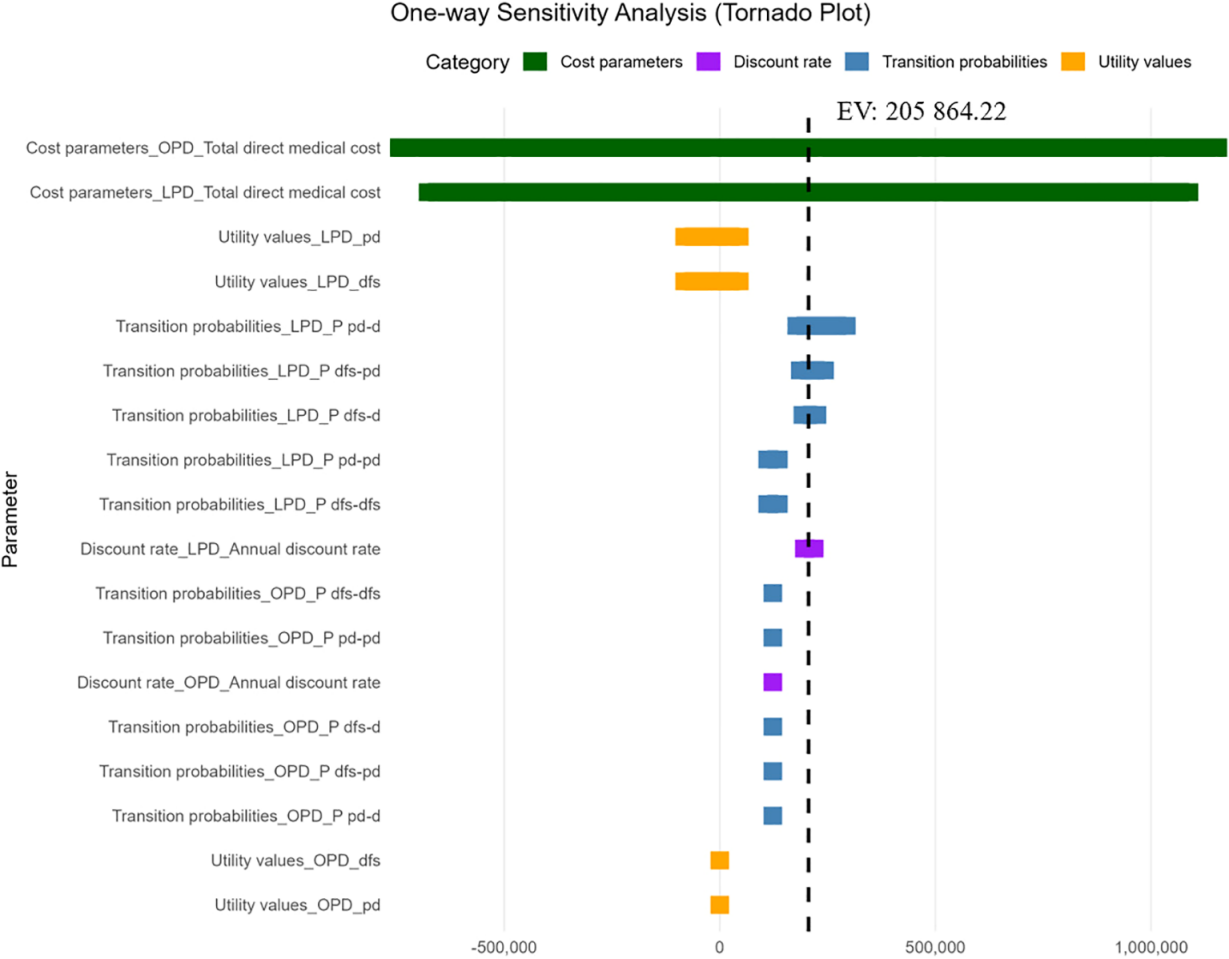
Storm chart of sensitivity analysis of OPD and LPD. OPD, open pancreaticoduodenectomy; LPD, laparoscopic pancreaticoduodenectomy.

The tornado diagram displayed the impact of each parameter’s variation within a specified range on the ICER value. The results show that the ICER is most sensitive to changes in the total direct medical costs for both the OPD and LPD groups. In contrast, variations in transition probabilities, utility values, and the discount rate exert relatively minor effects (**Figure 3**). However, regardless of the parameter changes, the ICER value remained below the WTP value, indicating that the model is relatively stable.

### 3.4 Probabilistic sensitivity analysis

**Figure 4:**
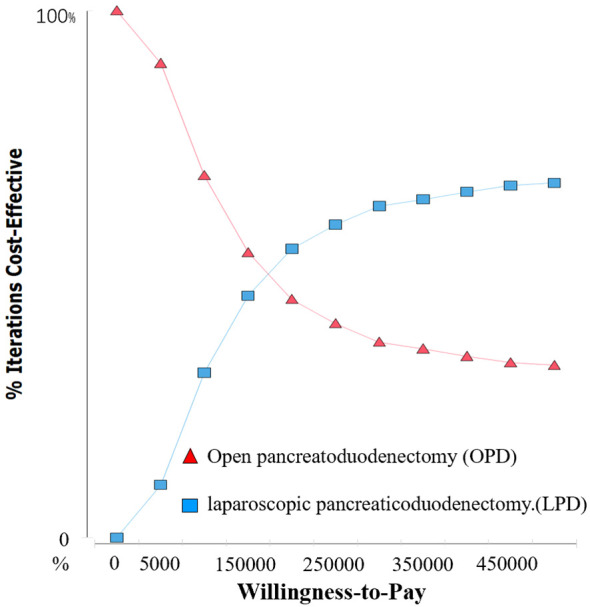
Cost acceptability curve of OPD and LPD.

As shown in **Figure 4**, under the condition that the WTP is less than 50,000 the probability that LPD has a cost-effectiveness advantage in China is 0. When the WTP is 200,000 LPD has a 50% probability of being more cost-effective than OPD. As the WTP value increases, the cost-effectiveness acceptability curve for LPD continues to rise, indicating a higher probability of having a cost-effectiveness advantage. This suggests that the model analysis results are relatively stable and have a high level of credibility.

## 4 Discussion

This study is the first to employ a Markov model using pancreatic ductal adenocarcinoma patients from a national regional medical center in China as a sample to conduct an economic benefit analysis of OPD and LPD. The results show that while LPD does not significantly outperform OPD in terms of survival extension, it remains an acceptable surgical option with certain cost-effectiveness for pancreatic cancer patients from a health economics perspective.

### 4.1 The clinical effectiveness of LPD is significant

Several clinical randomized controlled studies have compared the advantages and disadvantages of OPD and LPD in the treatment of pancreatic head malignancies, mostly focusing on early complications and survival rates. A recent prospective randomized controlled study published in JAMA Surgery included 200 patients with pancreatic ductal adenocarcinoma who underwent OPD and LPD surgery, with a 1:1 random grouping. The short-term results showed that although LPD surgery took half an hour longer than OPD (330.00 min *vs* 297.00 min, P < 0.001), it had less intraoperative bleeding (145.00 ml *vs* 200.00 ml, P = 0.020), a lower incidence of postoperative complications (46.00% *vs* 54.00%, P = 0.032), and the same median postoperative hospital stay for two groups (14.00 days *vs* 14.00 days, P = 0.210) (13). A meta-analysis and the original study reached similar conclusions that LPD has advantages over OPD in short-term clinical indicators, including shorter hospital stays, lower blood loss and transfusion requirements, and lower surgical site infections (13–16).

There is no significant difference in short-term clinical indicators between OPD and LPD, but few studies have analyzed the long-term survival data of OPD and LPD surgeries, with inconsistent results. A retrospective study by Zhou Haihua and colleagues analyzed the survival prognosis of 63 pancreatic cancer patients who underwent pancreatoduodenectomy, with a follow-up of 1 month to 5 years (29). The study showed that the total survival times for LPD (N = 29) and OPD (N = 34) were 27.46 months and 29.89 months, respectively (P < 0.031), and the disease-free survival times were 20.78 months and 19.86 months, respectively (P < 0.042). A randomized controlled study published in the Annals of Surgery in 2024, jointly completed by 14 large third-grade hospitals in China, compared the effects of open and laparoscopic pancreatoduodenectomy for pancreatic and periampullary tumors (30). The study included 656 patients, randomly divided into LPD and OPD groups at a 1:1 ratio. The results showed that the 3-year overall survival rates for LPD (N = 328) and OPD (N = 328) were 59.10% and 54.30%, respectively (P = 0.330). Another study by Kantor O and colleagues analyzed data from the National Cancer Data Base (NCDB) from 2010 to 2013 on patients who underwent OPD and LPD surgeries for pancreatic ductal adenocarcinoma. The results showed similar median overall survival times for LPD (N = 828) and OPD (N = 7385) of 20.70 months and 20.90 months, respectively (P = 0.680) (31).

The aforementioned studies indicate that LPD’s clinical outcomes are not inferior to OPD in terms of surgical complications or oncological outcomes. However, in clinical practice, implementing LPD requires higher costs than OPD. The treatment of pancreatic cancer is a long-term, multidisciplinary process, and treatment costs are an important factor that cannot be ignored, especially for middle and low-income populations. Excessive treatment costs may affect patients’ and their families’ decisions regarding treatment. This study included patients with pancreatic ductal adenocarcinoma who underwent LPD (N = 100) and OPD (N = 100) at the pancreatic surgery department of a national regional medical center in Western China between 2017 and 2020, with a follow-up period of 3.5 to 6.5 years. The results showed that the median overall survival time (24.00 months *vs* 22.00 months, P = 0.100) and the median disease-free survival time (15.00 months *vs* 14.00 months, P = 0.024) for OPD were not inferior to the LPD approach.

### 4.2 LPD has a cost-effectiveness advantage

Pancreatic cancer is highly malignant, progresses rapidly, and has a poor prognosis. Assessing the long and short-term clinical effects of OPD and LPD, and further evaluating the health economics indicators of these two surgical methods, will provide more comprehensive and enriched decision-making information for doctors and patients, facilitating the best decision-making. Previous economic studies on pancreatic cancer have mostly compared different chemotherapy regimens (32–35), and there are currently no economic evaluations of surgical options. Due to the high difficulty of LPD surgery, high equipment requirements, high technical demands on the surgeon, and high surgical costs, this study shows that the average direct cost per case of pancreatic cancer LPD is about 5000 yuan more expensive than OPD. A meta-study including 22 studies from six countries (mainly the United States, Italy, and the Netherlands) from 2008 to 2020 on the treatment costs of OPD and LPD showed that after integrating the economic levels of different countries and regions, the average cost of surgical instruments for LPD was $3402.10, the average cost of the operating room was $4484.13, and the average total cost of surgery was $6737.47 (36). While for OPD, the average cost of surgical instruments was $1992.92, the average cost of the operating room was $4255.57, and the average total cost of surgery was $5659.91 Our study, similar to previous results, indicates that LPD has higher cost expenses compared to OPD. This study constructed a Markov model based on the methods of pancreatoduodenectomy to explore the cost-effectiveness of the two main pancreatoduodenectomy methods. The results show that after 120 cycles, for each additional QALY, the ICER value was 205,864.22 yuan/QALY, which is lower than the WTP (268 074.00 yuan), indicating that LPD has a cost-effectiveness advantage from a health economics perspective.

### 4.3 Cost-effectiveness advantage of LPD: variability and influencing factors

Surgical approaches, societal willingness-to-pay (WTP), and other factors may all contribute to regional differences in cost-effectiveness. Our results show that the LPD has a slight cost-effectiveness advantage: the total cost of LPD increased by 13,175.31 yuan compared to OPD, with an incremental QALY of 0.063. A previous systematic review including 22 studies and 152,651 patients specifically evaluated the cost-effectiveness of minimally invasive versus open pancreatic resections, finding that minimally invasive procedures had higher total hospitalization costs than open surgery (€1379 ± 919, P<0.001), but provided an additional health benefit of 0.2 QALYs (36).Another Dutch multicenter study (N = 104) further confirmed the impact of WTP on cost-effectiveness: when WTP was €0, the probability of LPD being cost-effective was 56.6%; when WTP increased to€2000, this probability rose to 78.6% (26). Similar trends are seen in Chinese studies, where the OPD-TRIANGLE approach generated an additional 0.0402 QALY at an incremental cost of $1,501.83, and at a WTP threshold of $60,000/QALY, the probability of it being the preferred treatment was 52.8% (37). These studies suggest that LPD demonstrates a certain cost-effectiveness advantage in most scenarios. However, due to the lack of unified WTP thresholds across countries and the influence of factors such as healthcare resource accessibility, insurance policies, and surgeon experience, the cost-effectiveness advantage of LPD is not absolutely stable. This highlights the need for future large-scale, multicenter, localized studies to further validate its health economic value in specific medical environments.

### 4.4 Limitations

This study still has certain limitations: (i) Differences in healthcare systems, cultural backgrounds, and geographical regions can influence patients’ prioritization of different health status dimensions. This, in turn, affects how patients report symptoms and their tolerance thresholds for those symptoms. Given the lack of local studies on clinical outcome-related utility values for OPD and LPD pancreatic cancer patients in China, it is necessary for future research to focus on collecting and validating local utility data among Chinese pancreatic cancer patients. Such research would provide essential parameters for health economic analyses of different pancreatic cancer treatment strategies in China. (ii) Due to conditional limitations, this study only included patients from a national regional medical center in Western China, and the representativeness of the patients is somewhat limited. It is hoped that subsequent multicenter, multi-sample studies can be conducted to further improve the Markov model, making the economic evaluation more representative. (iii) Due to the limitations of retrospective studies, this study could not collect indirect and hidden costs, which were not included in this model. The maturity of the data needs further improvement, providing a new direction for future research. (iv) With the widespread application of large language model (LLM) algorithms in the medical field, deep learning models hold promise for investigating latent factors and enhancing the accuracy of economic evaluations (38–42). Among these, recurrent neural networks (RNNs) and their variants like long short-term memory (LSTM) and gated recurrent unit (GRU) architectures, offer significant advantages in modeling complex data. These capabilities are anticipated to yield deeper insights than traditional methodologies. However, the Markov model, as a classical health economic analysis method, can also yielded robust and credible findings in our study.

### 4.5 Clinical implications

Studies have shown that financial resources significantly influence patient’s surgical decision, adherence to cancer treatment and quality of life (43, 44). Therefore, financial status is a critical factor that should be considered when performing decision-making (45). Against the backdrop of the low survival rate and high treatment costs of pancreatic cancer, our study compared the economic benefit of OPD and LPD. The findings provide clinical evidence to improve surgical decision-making with respect to pancreatic cancer, empowering patients to make choices that align with their preferences and best interests.

## 5 Conclusion

Our study is the first to analyze the long-term clinical effects of OPD and LPD in pancreatic cancer patients. The results show that while LPD does not significantly extend survival, it has certain cost-effectiveness advantages from a health economics perspective. Because the incremental gain in quality-adjusted life years (QALYs) is small, it does not indicate a clinically meaningful extension of survival. Combined with previous studies showing the short-term safety and effectiveness of LPD, we believe that LPD remains an acceptable surgical option with certain cost-effectiveness for pancreatic cancer patients.

However, considering the generally low survival rates for pancreatic cancer and the fact that LPD is a highly technique-dependent surgical method, surgeons should still make careful choices between the two surgeries based on the patient’s expected survival time and financial situation.

## Data Availability

The raw data supporting the conclusions of this article will be made available by the authors, without undue reservation.
