# Supplementary figures and images for: Cost-effectiveness of open versus laparoscopic pancreaticoduodenectomy: a retrospective Markov model analysis from China
Source: Front Oncol. 2025 Dec 17;15:1616793. doi: 10.3389/fonc.2025.1616793 (PMC12753395; doi:10.3389/fonc.2025.1616793)

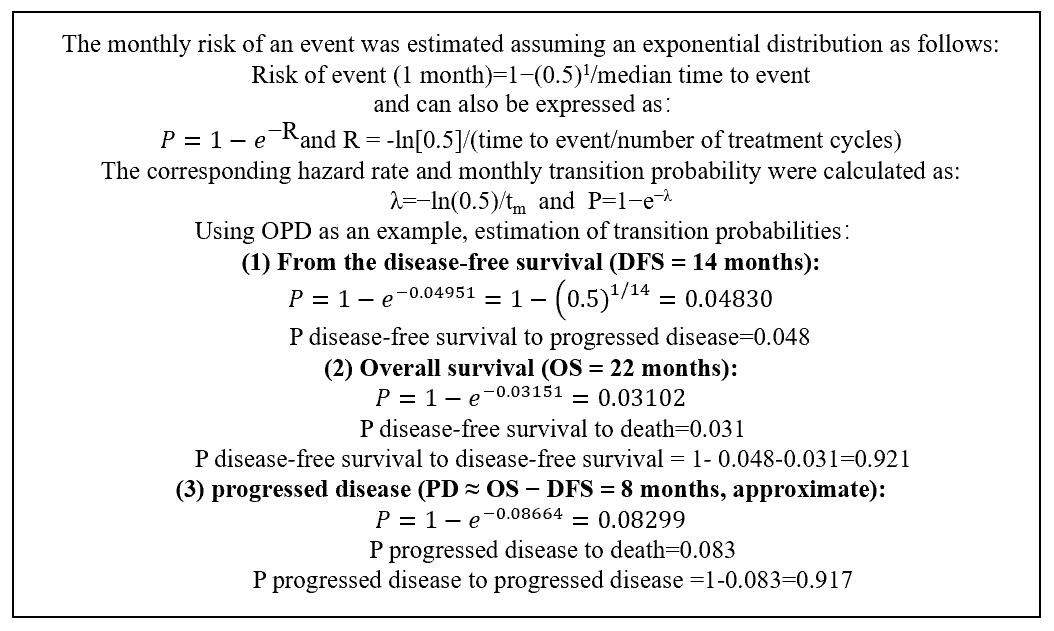

Supplement: Supplementary file 1 [file Image1.png]
