# Supplementary material for: Cost-effectiveness of open versus laparoscopic pancreaticoduodenectomy: a retrospective Markov model analysis from China
Source: Front Oncol. 2025 Dec 17;15:1616793. doi: 10.3389/fonc.2025.1616793 (PMC12753395; doi:10.3389/fonc.2025.1616793)
Supplement: Supplementary file 2 [file Table1.docx]

Supplementary Material

# Supplementary Table and Figure

## Supplementary Table S1

| **Table S1. Summary of key parameters and model assumptions** | | | | | | |
| --- | --- | --- | --- | --- | --- | --- |
| **Category** | **Parameter** | **OPD Baseline** | **LPD Baseline** | **Range (SA)** | **Distribution** | **Data source** |
| Model structure | Model type | Markov model | Markov model | — | — | — |
| Health states | Disease-free survival | — | — | — | — | — |
|  | Progressed disease | — | — | — | — | — |
|  | Death | — | — | — | — | — |
| Time settings | Cycle length | 1 month | 1 month | — | — | — |
|  | Time horizon | 10 years | 10 years | — | — | — |
|  | Half-cycle correction | Applied | Applied | — | — | — |
| Transition probabilities | P dfs-dfs | 0.921 | 0.927 | ±20% | Dirichlet | (20-21) * |
|  | P dfs-pd | 0.048 | 0.045 | ±20% | Dirichlet | (20-21) * |
|  | P dfs-d | 0.031 | 0.028 | ±20% | Dirichlet | (20-21) * |
|  | P pd-pd | 0.917 | 0.926 | ±20% | Dirichlet | (20-21) * |
|  | P pd-d | 0.083 | 0.074 | ±20% | Dirichlet | (20-21) * |
| Cost parameters  (RMB, 2023 values) | Total direct medical cost | 95 994.18 | 100 013.20 | ±20% | Gamma | HIS |
| Utility values | DFS | 0.792 | 0.810 | ±10% | Beta | (23-25) |
|  | PD | 0.650 | 0.650 | ±10% | Beta | (23-25) |
| Discount rate (%) | Annual discount rate | 5 | 5 | 0-8 | Fixed | (22) |

Note: * Supplementary Figure S1.

Abbreviation: dfs: disease-free survival; Pd: progressed disease; d: death; OPD: open pancreatoduodenectomy; LPD: laparoscopic pancreatoduodenectomy; SA: sensitivity analysis; HIS: hospital information system.

## 1.2 Supplementary Figure

## Figure S1：Transition probability derivation formula
